# Combined electrospun fibre-microneedle patches for enhanced transmucosal delivery of benzodiazepines and proteins

**DOI:** 10.1039/d6bm00216a

**Published:** 2026-05-27

**Authors:** Cerys Berry, Jake G. Edmans, Klaudia M. Slowik, Robert A. Byers, Simon Danby, Paul V. Hatton, Craig Murdoch, Helen E. Colley

**Affiliations:** a School of Clinical Dentistry, University of Sheffield 19 Claremont Crescent Sheffield S10 2TA UK paul.hatton@sheffield.ac.uk; b School of Mathematical and Physical Sciences, Brook Hill, University of Sheffield Sheffield S3 7HF UK; c Sheffield Dermatology Research, School of Medicine & Population Health, University of Sheffield Sheffield S10 2RX UK; d Insigneo Institute for Biomedical Engineering, University of Sheffield Sheffield UK

## Abstract

The oral mucosa is highly vascularised, which permits rapid drug uptake into the systemic circulation, avoiding first-pass metabolism for chemicals that penetrate the epithelial permeability barrier. Electrospun mucoadhesive patches have been developed for controlled drug delivery and although contact times and drug distribution are improved, transmucosal drug permeation is still limited to small lipophilic molecules. Incorporation of solid microneedles with an electrospun patch to physically disrupt the epithelial barrier whilst simultaneously delivering a payload, can overcome these issues whilst addressing the drug-loading limitations often experienced with microneedles. Here, we developed a mucoadhesive patch and microneedle composite for delivery of the benzodiazepine, midazolam hydrochloride, required for rapid, systemic delivery to treat status epilepticus and antigen-binding fragments (f(ab)), proteins too large to cross the epithelial permeability barrier. Solid polylactic acid microneedles were manufactured through reverse micromoulding, optimised for buccal permeation and imaged using optical coherence tomography. Electrospun mucoadhesive patches, loaded with midazolam hydrochloride or f(ab), were combined with the microneedles and permeation through tissue-engineered buccal mucosa quantified by high-performance liquid chromatography or immunoassay and imaged by fluorescence confocal microscopy. Microneedle-mediated patch delivery enhanced patch retention time, drug delivery rates and overall permeation when compared to patch-only controls, facilitating rapid delivery of time-sensitive midazolam within minutes, and a 15-fold increase in f(ab) permeation over 2 h, importantly delivering it through the epithelium into the underlying lamina propria. This study demonstrates that microneedle-mediated mucoadhesive patches can enhance transmucosal drug delivery for poorly-permeable drugs and holds significant potential when rapid, systemic drug delivery is required.

## Abstract 2

A microneedle mucoadhesive patch composite enhances drug delivery to the oral mucosa.

## Graphical abstract

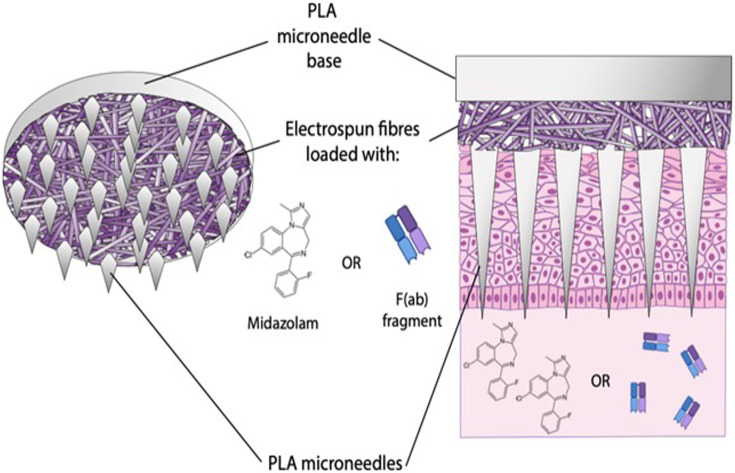

## 1. Introduction

Transmucosal drug delivery is an attractive alternative to parenteral and oral routes of administration as it avoids first-pass metabolism and gastrointestinal tract enzymatic degradation, reduces systemic side-effects and has a rapid on-set of action.^1^ This is particularly beneficial when delivering drugs with time-sensitive dosing regimens or low bioavailability. Additionally, unlike the more common oral administration, transmucosal medications can be administered to patients who are nil by-mouth or dysphagic. Although drug delivery by intravenous injection offers the greatest bioavailability, delivering the drug directly to the bloodstream or near the site of action, it is associated with limitations including needle fear in patients,^2^ decreased drug stability in solution compared to solid dosage forms, and the need for trained personnel during administration.

There are a number of potential sites within the oral cavity for oral transmucosal drug delivery, with the sublingual (under the tongue) and buccal mucosa being particularly suitable. This is because these sites display non-keratinised mucosal epithelium that is several-times more permeable than skin and a highly vascularised underlying connective tissue^3^ that facilitates rapid drug uptake into the systemic circulation.^4^ Although transmucosal delivery has been developed for some drugs (*e.g.* glyceryl trinitrate for angina, fentanyl citrate for pain, and prochlorperazine for nausea) there remains a shortage of formulations specifically developed for this route with medications often prescribed off-label due to the lack of options.^5,6^

Midazolam hydrochloride (HCl) used for the treatment of status epilepticus (a seizure lasting longer than five minutes or recurrent seizures without recovery of consciousness between them), requires immediate drug administration.^7,8^ Currently, midazolam HCl is delivered as a liquid by syringe application onto the oral mucosal surface between the gums and the buccal mucosa in order for it to be absorbed into the bloodstream through the mucosal tissue. This treatment is difficult to apply to the desired intraoral location in patients often experiencing convulsions, is rapidly washed away by saliva and is often swallowed accidentally leading to inadequate dosing, risk of off-target toxicity and reduced drug efficacy. This is a significant problem, especially when rapid and effective drug delivery is paramount. These problems may be circumvented if midazolam HCl was delivered *via* a mucoadhesive device that adheres tightly to the oral mucosa upon application, allowing continued and controlled drug release directly to the epithelium and into the bloodstream.

The advent of polymer-based fibrous membrane formulations prepared using electrospinning has made a significant advancement in the field of mucosal drug delivery.^9–11^ The use of mucoadhesive polymers coupled with a large surface area facilitates tissue adhesion while their flexibility allows conformity to curved surfaces, leading to prolonged tissue residence times.^9^ Incorporation of a number of drugs has been achieved,^12–14^ with recent successful translation to clinical use for delivery of clobetasol propionate^15^ and tacrolimus.^16^ However, so far, the technology has been limited to small lipophilic molecules that easily permeate the oral epithelium at a sufficient rate. Although peptides and proteins have been successfully incorporated,^10,17^ their large molecular mass means that they do not easily traverse the permeability barrier of intact oral epithelium and current formulations are more suitable for delivery to lesional tissue where the permeability barrier is compromised.^18^

Microneedles are physical permeability enhancers that act by piercing the epithelium, allowing drugs to bypass the permeability barrier, aiding passive diffusion into the tissue and microcirculation.^19^ Due to their micro-size, these needles are minimally invasive and are well-received by patients.^20^ Previous studies have demonstrated transmucosal delivery using drug-loaded solid or dissolvable microneedles for enhanced delivery to the oral mucosa of small molecules including anti-inflammatory steroids,^21^ anaesthetics,^22^ and chemotherapeutics for the local treatment of oral carcinomas^23^ or oral potentially malignant disorders.^24^ Larger biomolecules including human insulin and human growth hormone have been delivered to the oral mucosa using dissolvable polyvinylpyrrolidone (PVP) and sorbitol microneedles,^25^ whilst ovalbumin and human immunodeficiency virus antigens have been delivered using coated, steel microneedles.^26^

More recently, microneedles have been developed as composites, encompassing a membrane for enhanced drug delivery. Zhang *et al.*, coupled biodegradable gelatin methacryloyl microneedles with a gelatin membrane for tetracycline and cytokine (IL-4 and TGF-β) delivery for periodontal tissue regeneration.^27^ Whilst others have looked at delivering synthetic corticosteroids alone^28^ or in combination with growth factors^29^ and antibacterials^30^ for the treatment of oral ulcers and oral submucous fibrosis using a range of biomaterials including silk fibroin, hyaluronic acid and hydroxypropyl trimethyl ammonium chloride chitosan. Creighton *et al.*, used integrated electrospun needles, manufactured from a range of synthetic polymers, loaded with ovalbumin to model vaccine delivery.^31^ However, as yet, microneedle-mediated delivery of antibodies to the buccal mucosa has not been attempted.

In this study, we explore new ways to administer drugs that are currently difficult to deliver, either because they are currently in liquid form (*e.g.* midazolam HCl) or that their high molecular mass prevents their permeation through the oral epithelium (*e.g.*, antibody for biotherapeutics). Using *ex vivo* porcine or tissue-engineered human oral (buccal) mucosa equivalents (OME), we show that an electrospun fibre-microneedle composite (EF-MN) displays high buccal adhesion, and facilitates rapid and controlled drug delivery of both small molecules and high molecular mass proteins directly to and through oral mucosa. This device holds great potential to transform oral mucosal drug delivery, opening up new avenues for treatment.

## 2. Materials and methods

### 2.1 Materials

All reagents were purchased from Merck (Gillingham, UK) unless otherwise stated. Polyvinylpyrrolidone (PVP; MW 2000 kDa) and Eudragit RS100 (MW 32 kDa) were kindly donated by BASF (Cheadle Hulme, UK) and Evonik Industries AG (Essen, Germany), respectively.

### 2.2 Fabrication of polylactic acid microneedles

Microneedle template moulds were generated using polydimethylsiloxane (PDMS; Sylgard 184, Dow Corning, Michigan, USA) by reverse moulding of microneedle master arrays (∅ 10 mm, 42 needles) with varying needle length (small; 300 μm, medium; 500 μm and large 750 μm). Briefly, PDMS (elastomer base : curing agent; 10 : 1 w/w) was mixed by centrifugation at 3000*g* for 2 minutes. The microneedle template was submerged in the PDMS contained within a moulded aluminium receptacle and degassed in a desiccator vacuum for 30 minutes before curing on a hot plate at 100 °C for 1 h. To melt cast polylactic acid (PLA; Ultimaker, Utrecht, Netherlands) microneedles, 0.2 g of PLA was added into each mould and placed in an oven at 200 °C for 1 h. The microneedles were cooled at room temperature, before removal from the moulds. PLA microneedle mechanical robustness was measured using the Vickers hardness (HV) test using a calibrated instrument (Foundrax Engineering Ltd, UK) with a square-based pyramid indenter and an applied load of 0.3 kg at 5 s dwell time. Three indentations were performed for each sample, with a distance of 2.5 times the diagonal length of the indentation used between measurement points. The diagonal length (D1 and D2) of a square indentation was measured using a scaled light microscope. To determine the HV number, the mean ± SD of three points of indentation was calculated for each of three independently manufactured samples, and calculated using the following equation:
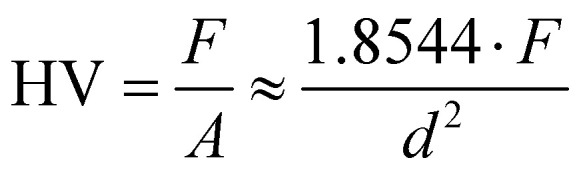
where *F* is the load in (kgf) and *d* is the mean diagonal length (mm).

### 2.3 Electrospinning and fabrication of mucoadhesive electrospun fibres

Electrospun fibres (EF) were fabricated using a system comprising a PHD2000 syringe pump (Harvard Apparatus, Cambridge, UK) and an Alpha IV Brandenburg power source (Brandenburg UK Ltd, Worthing, UK) as previously described.^11^ Plastic syringes (1 mL volume; Henke Sass Wolf, Tuttlingen, Germany) were used to drive the solutions into a 15-gauge blunt metallic needle (Fisnar Europe, Glasgow, UK). Non-medicated mucoadhesive EF were produced as previously described^11^ from solutions containing 10% w/w PVP and 12% w/w Eudragit RS100 in 97% v/v ethanol and 3% v/v water. Briefly, PVP and Eudragit RS100 were added to ethanol and mixed at room temperature using a magnetic stirrer until dissolved. Electrospinning was performed at room temperature, 45% humidity at 19 kV, a flow rate of 2 mL h^−1^ and a flight path of 14 cm, utilising a flat collector. For the midazolam HCl-loaded EF, 5% w/w midazolam HCl (Tocris Bioscience, Bio-Techne, Minneapolis, USA) was added to the polymers and mixed until homogenous (and herein referred to as MDZ@EF). To yield MDZ@EF, with a therapeutically relevant dose of midazolam HCl, the flight path was reduced to 7 cm. For incorporation of antibody f(ab) fragments, biotinylated goat f(ab) anti-mouse IgG and Texas Red-conjugated goat f(ab) anti-mouse IgG (abcam, Cambridge, UK) were incorporated into the EF (f(ab)@EF) as previously described.^18^ Briefly, 10% w/w PVP and 12% w/w Eudragit RS100 were dissolved in 100% ethanol overnight. Immediately before electrospinning, 90 µL f(ab) antibody (1 mg mL^−1^ in 0.01% sodium azide, 1% bovine serum albumin, 0.87% sodium chloride, 0.42% tripotassium orthophosphate) was added to the polymers, mixed for 60 s and then electrospun using the parameters above. The resultant f(ab)@EF contained 0.013% f(ab), w/w and maintained the same 97% ethanol solvent conditions as the non-medicated patch at the time of electrospinning. To visualise the homogeneity of drug distribution within the f(ab)@EF, 0.2 mL of Texas Red-conjugated f(ab) polymer (0.013%; w/w) was electrospun directly onto a glass coverslip to create a thin layer of distinguishable fibres. The coverslip was sealed onto a microscopy slide and visualised under confocal fluorescence microscopy (Nikon spinning confocal microscope, Tokyo, Japan).

### 2.4 Combination of microneedles with mucoadhesive electrospun fibres

**Fig. 1:**
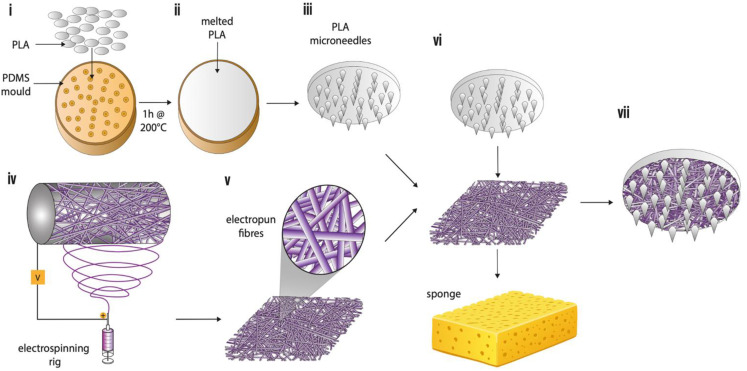
A schematic diagram detailing the manufacturing process for the electrospun fibre-microneedle composite. (i) PLA was loaded into a PDMS negative template and heated in an oven to 200 °C for 1 h. (ii) The microneedles were cooled at room temperature to allow them to solidify, (iii) then removed from the PDMS mould. (iv) Electrospun fibres were produced by spinning PVP (10%, w/w) and RS100 (10%, w/w) (v) with f(ab) (0.013%) or midazolam (5%, w/w) incorporated. (vi) The microneedles were then submerged in 2 mL deionised H_2_O with excess water removed using a sponge and the microneedles attached to the electrospun fibres by applying gentle pressure for 5 s. (vii) The final electrospun fibre-microneedle composite device showing the microneedles with electrospun fibres and base plate.

To generate an electrospun fibre-microneedle composite (EF-MN), the PLA microneedle array was submerged into 2 mL of deionised water, and excess removed with a sponge, to create a moist surface. A disc (∅ 10 mm) of EF was placed face down onto a dry sponge, and the microneedle array inserted through the basolateral surface for 3 s with gentle pressure applied so it pressed firmly against the PLA baseplate (Fig. 1).

### 2.5 Scanning electron microscopy

Electrospun or microneedle samples were mounted on 25 mm aluminium stubs and gold sputtered for 90 s with a 30 mA current. A Tescan Vega3 LMU scanning electron microscope (SEM, Tescan Orsay Holding, Brno, Czechia) was used to image samples using an emission voltage of 10 kV. The lengths and widths of 54 small, medium and large microneedles we measured using image J software (National Institute of Health, Bethesda, Maryland, USA). Fibre diameter of electrospun fibres was measured using ImageJ Software. Each fibre was randomly selected through generation of coordinates on a superimposed grid on an image with 10 fibres measured on three independent images for three independent membranes.

### 2.6 Physicochemical characterisation of electrospun fibres

An electronic digital balance was used to measure EF mass while thickness was measured at three randomly selected points using Vernier callipers. To determine swelling, EF (∅ 10 mm) were weighed and submerged in 2 mL PBS for 2 h. Excess water was removed using a paper towel, and the EF re-weighed to quantify percentage swelling. To measure residency time, *ex vivo* porcine buccal mucosa was cut (3 × 5 cm) and attached to tissue culture plates. Non-medicated EF, microneedles alone, and EF-MN (∅ 10 mm) were attached to the mucosa with 20 µL of PBS and gentle pressure for 5 s and the wells filled with 1 mL of PBS and placed on an orbital shaker at 250 rpm at 37 °C and monitored for up to 180 h and the detachment time recorded.

### 2.7 Release profile of midazolam HCl from the electrospun fibres using reverse phase high-performance liquid chromatography

Three independently prepared EF (∅ 10 mm) containing midazolam (MDZ@EF; 5%, w/w) were weighed and submerged in 2 mL PBS in a 12-well plate at 250 rpm at 37 °C and 100 μL samples collected at 5, 15, 30, 60, and 120 minutes. Each 100 μL sample taken was replaced with the equivalent volume of fresh PBS. Midazolam HCl concentrations were detected through reverse phase high-performance liquid chromatography (RP-HPLC) (Shimadzu Corporation, Kyoto, Japan) and UV detection using a XBridge BEH-C18 column (4.6 mm × 250 mm; 130 Å pore size, Waters Corporation, Milford, Massachusetts, USA) with a mobile phase of 30% acetonitrile and 70% water containing 0.1% trifluoroacetic acid (v/v) and a flow rate of 1 mL min^−1^ with an injection volume of 10 µL. Midazolam HCl was detected at a wavelength of 245 nm and exhibited a retention time of 10.5 minutes. 1-Hydroxymidazolam (Cayman Chemical, Ann Arbor, MI, USA) peak was obtained at 8.5 minutes retention. Sample HPLC area peaks were compared to a standard curve. The method was validated in terms of linearity and precision using standards ranging from 30 to 0.98 µg ml^−1^ and 10 to 0.63 µg ml^−1^ for midazolam HCl and 1-hydroxymidazolam, respectively. Accumulated release of drug was calculated from measured sample concentrations using eqn (1) below. At each timepoint (*T*) the percentage release is the mass (*M*) permeated (present in PBS and lost to previous sampling) divided by the applied dry mass fraction of drugs in the electrospinning solution. Mass is related to concentrations (*C*) and volumes (*V*) *via* standard molar relations.1
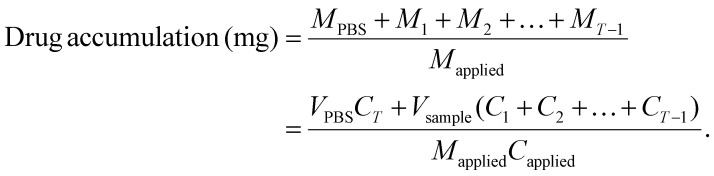


Percentage release was calculated from measured sample concentrations using eqn (2).2
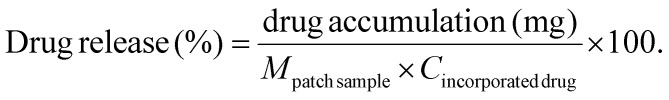


### 2.8 Generation of tissue-engineered oral mucosal equivalents

Immortalised human buccal keratinocytes (FNB6 hTERT; Ximbio UK) were cultured in flavin- and adenine-enriched medium as previously described.^32^ Full-thickness tissue-engineered human OME were constructed as previously described.^33^ In brief, rat tail type I collagen (1 mL) was added to 12-well, 0.4 μm pore transwell ThinCerts™ (Greiner Bio One Ltd, Stonehouse, UK) and left to set at 37 °C in a humidified atmosphere for 30 minutes. Once set, FNB6 were seeded topically at a density of 5 × 10^5^ cells in 500 µL of flavin- and adenine-enriched medium and cultured submerged. After 48 h, OME were raised to an air-to-liquid interface and cultured for a further 10 days, with the medium changed every 2–3 days.

### 2.9 Midazolam HCl permeation through tissue-engineered and *ex vivo* buccal mucosa

MDZ@EF without or with microneedle (MDZ@EF-MN), were applied to OME at 37 °C and 250 rpm. The MDZ@EF and MDZ@EF-MN were applied for 2 h and samples taken at 5, 15, 30, 60, and 120 minutes and permeation percentage quantified by RP-HPLC as previously described. For investigations using Franz diffusion cell system (PermeGear Inc., Hellertown, Pennsylvania, USA), *ex vivo* porcine buccal mucosa was cut to 1 mm thickness using a dermatome and 2.5 cm^2^ sections mounted with the epithelium facing the donor chamber. 5 mL PBS was maintained in the receptor chamber with gentle mixing at 37 °C. MDZ@EF and MDZ@EF-MN were applied to the apical surface and after 2 h, 200 µL of solution was removed from the donor chamber and permeation calculated using RP-HPLC as previously described To measure the amount of drug within the pig mucosa, mucosa pieces were first heated to 65 °C for three minutes to enable removal of the epithelium, which was subsequently cut into smaller pieces and placed in 1 mL acetonitrile and treated with ultrasound for 10 minutes before filtering with a 0.22 µm cellulose acetate filter for HPLC analysis as described previously.

### 2.10 Release profile of biotinylated f(ab) antibodies using an enzyme-linked immunosorbent assay

Three independently prepared biotinylated f(ab)-loaded EF (f(ab)@EF) (∅ 10 mm) were weighed and submerged in 2 mL PBS in a 12-well plate, previously blocked overnight with BSA (10% w/v), at 250 rpm at 37 °C and 100 μL samples collected at 5, 15, 30, 60, and 120 minutes. Each 100 μL sample taken was replaced with the equivalent volume of fresh PBS. Released f(ab) was quantified over time using a modified ELISA as previously described.^17,18^ Briefly, IgG from mouse serum was diluted in PBS to a concentration of 10 µg mL^−1^ and 100 µL added to each well of a high binding 96-well plate and incubated at room temperature overnight. The plate was washed three times with 0.05% Tween-20 in PBS before blocking with 300 µL BSA (1% w/v) at 4 °C overnight. Wells were washed again, and 100 µL of each sample added for 2 h at room temperature. After washing, a 1 : 40 dilution of horseradish peroxidase (HRP)-conjugated streptavidin was produced in PBS and 100 µL added to each well, to bind to any biotinylated antibody sites. After a final wash, 100 µL 3,3′,5,5′ tetramethylbenzidine (TMB) was added to each well and left to develop colour for 20 minutes. To stop the reaction, 50 µL of 1 M hydrochloric acid was added to each well. The optical density was measured at 450 nm using a spectrophotometer.

### 2.11 Visualisation of microneedle penetration into porcine oral mucosa using optical coherence tomography

Optical coherence tomography (OCT; VivoSight, Michelson Diagnostics, Maidstone, UK) was used to visualise microneedle penetration in real time and to monitor pore closure following microneedle removal. Imaging was performed at an acquisition rate of 20 kHz using a 1305 nm Axsun laser (Δ*λ* = 147 nm), enabling tissue visualisation to a depth of 1–2 mm. A scan area of 4 × 4 mm (*X* × *Y*) was used, with lateral and axial pixel sizes of 20 µm and 3.91 µm, respectively. Time-lapse imaging, consisting of 20 repeat scans, was used to assess micropore closure over a 5 minute period.

### 2.12 Permeation quantification of fragmented antibodies through tissue-engineered oral mucosal equivalents

F(ab)@EF and with microneedle incorporation (f(ab)@EF-MN) were manufactured with biotinylated f(ab) and applied to OME for 2 h with 1 mL of PBS placed in the basal chamber. 100 µL samples were removed from the basal chamber at 5, 15, 30, 60 and 120 minutes and replaced with fresh PBS. Permeated f(ab) fragment was quantified using an ELISA as previously described.

### 2.13 Visualisation of microneedle-mediated Texas Red-conjugated f(ab) permeation through tissue-engineered oral mucosa equivalents

Texas red-conjugated f(ab)@EF and f(ab)@EF-MN were manufactured as previously described. F(ab)@EF and f(ab)@EF-MN were applied to OME for 2 h and then fixed in formalin for 24 h at 4 °C. The models were removed from the transwell and placed epithelium-down onto glass bottom confocal dishes. The f(ab) was detected under fluorescent confocal microscopy (Nikon W1 spinning disk confocal, Tokyo, Japan).

### 2.14 Histological analysis

Human gingival mucosa biopsies were collected from the Charles Clifford Dental Hospital, Sheffield Teaching Hospitals NHS Foundation Trust with written, informed consent form donors undergoing routine dental surgery (ethical approval reference 09/H1308/66) or for buccal tissue, from healthy volunteers (ethical approval reference UREC 043074). Floor of the mouth (FOM) histological sections were sourced from archived patient samples (ethical approval reference 22/SC/0111). *Ex vivo* porcine mucosa, acquired from a local abattoir, were isolated from the gingival, buccal and floor of the mouth regions, and stored in DMEM culture medium or at −20 °C, then thawed at room temperature when required. For histological analysis, all samples, including OME, were fixed in 10% buffered formalin overnight at 4 °C, and processed using a benchtop tissue processing machine (Leica TP1020, Leica Microsystems, Wetzlar, Germany). Samples (5 µm) were stained with haematoxylin and eosin (H&E) using a linear staining machine (Leica ST 4040, Leica Microsystems, Lussloch, Germany) and mounted using dibutyl phthalate polystyrene xylene (DPX) before imaging using an Olympus BH-2 microscope and associated Olympus SC50 camera and CellSens Entry imaging software (Evident Europe GmbH, Hamburg, Germany). Epithelium thickness was determined using ImageJ (National Institute of Health, Bethesda, Maryland, USA), by measuring the distance between the top and bottom of the epithelium at three randomly selected locations from three independent biopsies.

### 2.15 Immunohistochemistry

Immunohistochemistry (IHC) staining was performed to determine the presence of CYP3A4 enzymes within OME and normal oral human buccal biopsies (ethical approval number: 003463). Formalin-fixed, paraffin-embedded samples were sectioned to 5 µm and mounted on adhesive glass slides (Superfrost Plus, Epredia Portsmouth, New Hampshire, USA). Antigen retrieval was performed using citrate buffer (pH 6) in a 2100 Antigen Retriever (Aptum Biologics Ltd, Hampshire, UK). The samples were incubated in serum-free protein block (Agilent Technologies, Santa Clara, California, USA) for 30 minutes at room temperature followed by a 1 h incubation in primary antibody directed to CYP3A4 (EPR6202; abcam, Cambridge, UK) at a 1 : 200 dilution. Negative control samples were incubated in PBS only. Sections were washed in PBS, followed by biotinylated secondary antibody incubation for 1 h, followed by a 30 minutes incubation with an avidin–biotin complex kit (Vector Laboratories, Newark, California, US) and antigens visualised using 30-diaminobenzidine tetrahydrochloride (DAB; Vector Laboratories, Newark, California, US), according to the manufacturer's instructions. Counterstain of the nuclei was performed using haematoxylin, followed by the dehydration of the section and mounting with Dibutylphthalate Polystyrene Xylene. Slides were imaged under light microscopy (Olympus Corporation, Japan).

### 2.16 Data analysis

All data and statistical analyses were performed using GraphPad Prism 9.4.1 software (La Jolla, San Diego, California, USA). All data was checked for normality using the Shapiro–Wilk Test. Significance between two data sets was determined using unpaired Student's *t*-test, or paired Wilcoxon sign rank test for elapsed data sets. To determine significance between multiple data sets, ordinary one-way or two-way ANOVA were employed followed by a Tukey's *post-hoc* test for multiple comparisons. Data is presented as mean ± standard deviation or median ± interquartile ranges and statistical significance assumed at *p < 0.05, **p < 0.01, and ***p < 0.001. The number of independent experiments performed (*n*) is detailed in the corresponding figure legends.

## 3. Results

### 3.1 Microneedle manufacture with optimal length for penetration through the oral mucosa

**Fig. 2:**
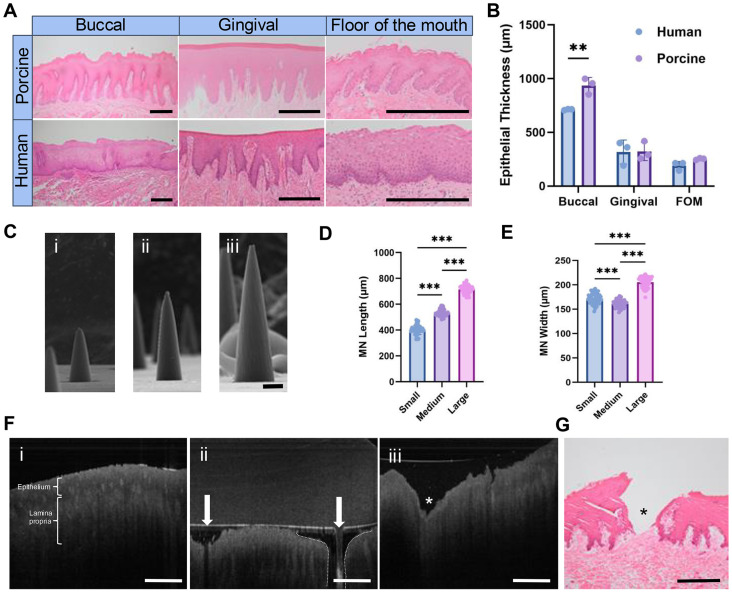
Microneedle manufacture with optimal length for penetration through the oral mucosa. (A) Representative histological images of H&E-stained human and porcine oral mucosa taken from three intraoral locations; buccal, gingival and floor of the mouth (FOM), scale bar = 400 μm. (B) The epithelial thickness was measured for each location; *n* = 3. (C) PLA microneedles (MN) were produced by reserve moulding from small (300 µm), medium (500 µm) and large (750 µm) template microneedles and visualised using scanning electron microscopy, scale bar = 100 µm, and (D) the microneedle length and (E) width measured; *n* = 54. Penetration visualisation using optical coherence tomography of the *ex vivo* porcine mucosa (Fi) before, (Fii) during (microneedle indicated with arrow and pore by dotted line) and (Fii) after microneedle application (pore indicted by *), scale bar = 500 µm. H&E image of the pore (*) created following removal of the microneedles (G), scale bar = 400 µm. Data are presented as mean ± SD with a statistically significant difference determined using a one-way (D and E) or two-way (B) ANOVA with *post hoc* Tukey's multiple comparisons where **<0.01, ***<0.001.

For localised delivery of biologics through the epithelial permeability barrier or for rapid delivery of small molecules to blood vessels for systemic delivery, microneedles need to penetrate the epithelium and basement membrane into the upper lamina propria, where the major capillary network resides. To determine optimum microneedle dimensions, histological analysis of human and porcine mucosa was performed at three intraoral locations (buccal, gingival and FOM). H&E staining revealed architectural differences between each area of the oral cavity and also inter-species variation (Fig. 2A). Porcine buccal mucosa displayed surface keratinisation, were significantly thicker (935 ± 75 μm *versus* 712 ± 5 μm; *p* < 0.001) and contained more rete ridges than human buccal mucosa (Fig. 2A and B). Porcine gingiva was more keratinised than human gingiva but the thickness of the epithelium was similar (322 ± 86 μm and 317 ± 112 μm for porcine and human, respectively) as were the level of rete ridges (Fig. 2A and B). For FOM, both mucosae were non-keratinised and of similar thickness (252 ± 8 μm and 190 ± 40 μm for porcine and human, respectively), although porcine FOM contained more rete ridges (Fig. 2A and B). The different thicknesses of epithelium between oral mucosal sites means that different size microneedles would be required for each tissue.

Manufactured by reverse moulding and melt casting of PLA, SEM analysis confirmed the production of high-quality microneedles with defined three-dimensional (3D) shapes and sharp tips (Fig. 2C). The resultant microneedle lengths were 401 ± 36, 534 ± 27 and 715 ± 30 µm (Fig. 2D) with mean widths of 172 ± 11, 161 ± 8, and 206 ± 11 µm, respectively (Fig. 2E) and a Vickers Hardness number of 15.98 ± 0.78 HV. OCT imaging of large microneedles *in situ* revealed that they were able to penetrate porcine buccal epithelium (Fig. 2Fi) and five minutes post-removal a pore was still visible (Fig. 2Fiii). Successful penetration and epithelium disruption was further verified by imaging of H&E-stained tissue fixed immediately following microneedle removal, which confirmed that the generated micropores penetrated through the epithelium into the underlying lamina propria (Fig. 2G).

### 3.2 Midazolam HCl-loaded mucoadhesive electrospun fibres fabrication and characterisation

**Fig. 3:**
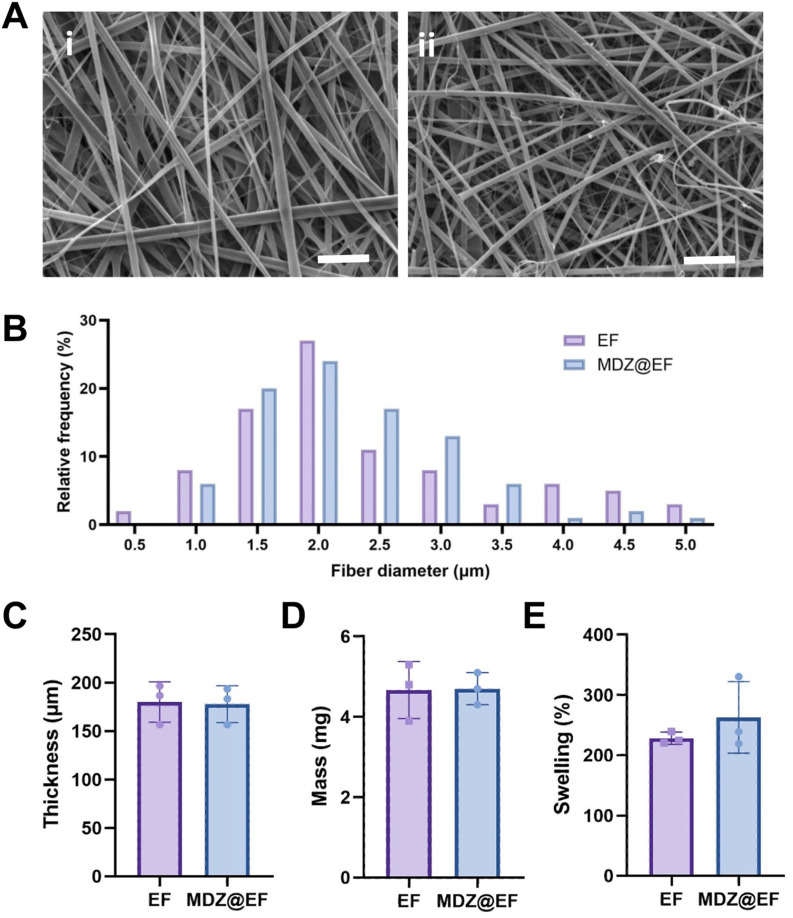
Midazolam-incorporated electrospun fibre manufacture and characterisation. Electrospun fibres (EF) manufactured from electrospinning PVP (10%, w/w) and RS100 (12%, w/w) were produced to incorporate midazolam hydrochloride (5% w/w) (MDZ@EF). Representative SEM images of the (Ai) non-mediated EF and (Aii) MDZ@EF revealed uniform (B) fibre diameters between the two EF with no significant difference in (C) thickness, (D) mass or (E) swelling properties. Scale bar = 20 µm. Data are presented as mean ± SD; *n* = 3.

To incorporate midazolam HCl at a therapeutically relevant dose, EF were manufactured to include 5% (w/w) midazolam HCl (MDZ@EF) with non-medicated EF serving as controls. SEM images revealed the presence of a network of microfibres for each sample (Fig. 3Ai and ii) with no statistical difference in diameter between the MDZ@EF (2.3 ± 0.5 µm) or non-medicated (2.3 ± 0.7 µm) EF (Fig. 3B). Incorporation of midazolam HCl did not alter the other physicochemical properties investigated. The thickness was 180 ± 21 µm and 178 ± 19 µm for the non-medicated EF and MDZ@EF membranes (Fig. 3C), with 10 mm ∅ membranes having a mean mass of 4.67 ± 0.71 mg and 4.70 ± 0.40 mg, respectively (Fig. 3D). The final midazolam HCl content of MDZ@EF 10 mm ∅ membranes was calculated as 235 ± 20 μg (equivalent to 5% w/w). Following 2 h submersion in PBS the mean swelling of MDZ@EF was 262 ± 9%, not significantly different when compared to non-medicated EF controls, 228 ± 10% (Fig. 3E).

### 3.3 An electrospun fibre-microneedle composite device increases the residency time to *ex vivo* oral mucosa

**Fig. 4:**
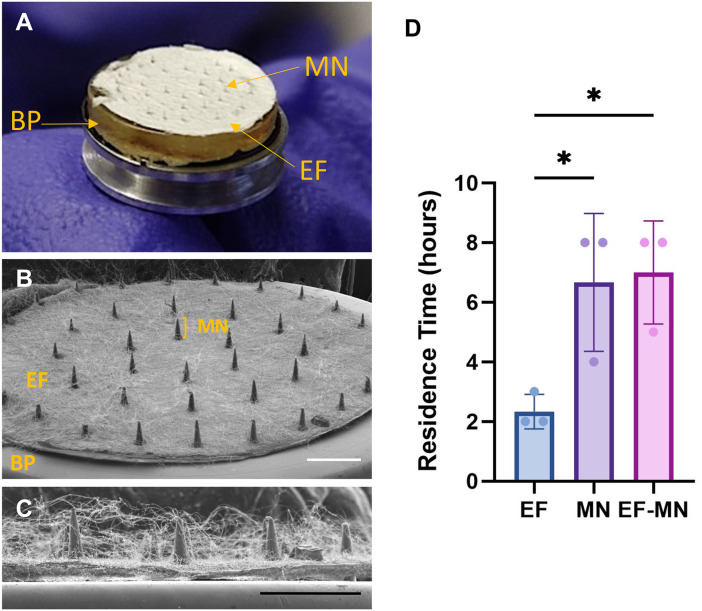
Electrospun fibre-microneedle composite patch. (A) An image of the electrospun fibre-microneedle (EF-MN) composite device showing the baseplate (BP), electrospun fibres (EF) and microneedles (MN) (B and C) with the ultrastructure revealed by scanning electron microscopy, where the microneedles can be seen penetrating through the EF. Scale bar = 2 mm and 1 mm, respectively. (D) Residence time of the EF recorded using *ex vivo* porcine buccal mucosa was enhanced when microneedles were present, comparable to applying the microneedles alone. Data are presented as mean ± SD with a statistically significant difference determined using a one-way ANOVA with *post hoc* Tukey's multiple comparisons where *<0.05; *n* = 3.

When combined together as an electrospun fibre-microneedle composite (EF-MN), the microneedles can clearly be seen protruding through the EF (Fig. 4A–C). When applied to *ex vivo* porcine buccal mucosa, residency time significantly (*p* < 0.05) increased from 2.3 ± 0.6 h for EF alone to 6.7 ± 2.3 h for MN alone and 7.0 ± 1.7 h for EF-MN (Fig. 4D).

### 3.4 Midazolam delivered from the electrospun fibres-microneedle composite increases the rate of permeation through oral mucosa

**Fig. 5:**
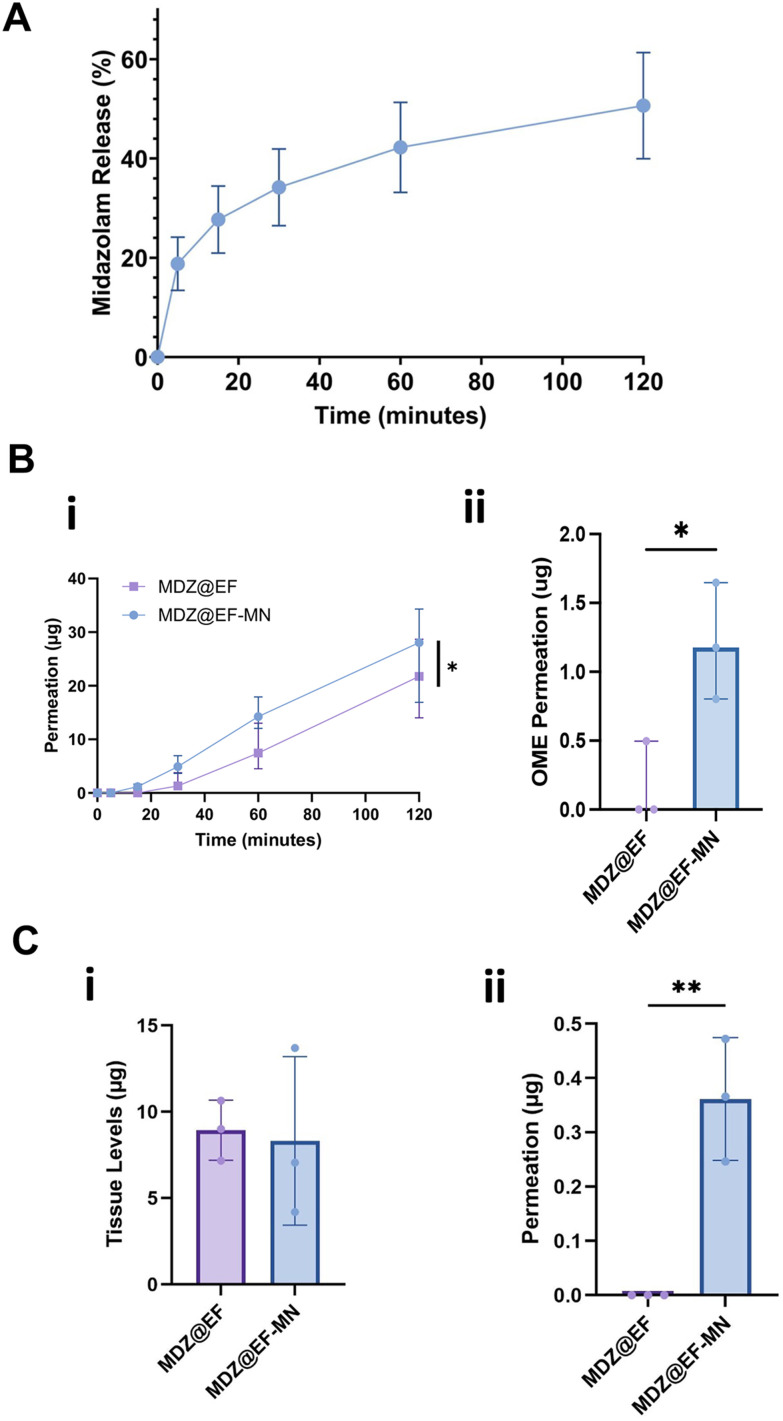
Midazolam release and permeation through oral mucosa is enhanced by microneedle-mediated delivery. Midazolam-incorporated electrospun fibres (MDZ@EF) were manufactured from PVP (10% w/w) and RS100 (12% w/w) with midazolam (5% w/w). (A) Midazolam release into PBS was measured over 2 h using RP-HPLC. (Bi) Permeation of midazolam from MDZ@EF and MDZ@Ef-MN across tissue-engineered oral mucosal equivalents (OME) was measured over 2 hours in static conditions (Bii) with a statistically significant difference in permeation after 15 minutes. (Ci) Retention within and (Cii) permeation through *ex vivo* porcine oral mucosa over 2 h using a Franz diffusion cell system. Data are presented as median ± quartile ranges with statistical analysis determined using a Wilcoxon matched paired signed-rank test (Bi) and Wilcoxon paired *t*-test (Bii) for non-parametric data and mean ± SD using a Student's *t*-test for parametric data (Ci and ii) where *p < 0.05, **p < 0.01; *n* = 3.

The incorporated midazolam HCl was rapidly released from the MDZ@EF into PBS with 18.8 ± 5.4% being released within the first 5 minutes before slowing to a steady release rate, with 51 ± 11% midazolam released after 2 h (Fig. 5A). Utilising OME to assess drug permeation, there was an overall significant increase in the bioavailability of midazolam HCl from the MDZ@EF-MN compared to EF alone over a 2 h period (*p* < 0.05), with 1.17 increase at 15 minutes (*p* < 0.05) (Fig. 5Bi and ii), a clinically relevant timeframe for effective treatment of status epilepticus where speed of drug delivery is paramount. Microneedle-mediated membrane delivery of midazolam HCl (MDZ@EF-MN) was also quantified through *ex vivo* porcine buccal mucosa using a Franz cell diffusion system. After 2 h, the amount of midazolam HCl recovered from within the buccal mucosa following application of microneedle-mediated membranes was similar to membranes alone; 8.3 ± 4.9 µg and 8.9 ± 1.7 µg (Fig. 5Ci). However, 0.4 µg (quartile ranges of 0.25 and 0.47 µg) midazolam HCl permeated through the tissue into the receptive medium when delivered from the microneedle-mediated membrane, whereas no detectable midazolam HCl was found after application of membrane alone (MDZ@EF) (*p* < 0.01) (Fig. 5Cii).

### 3.5 Midazolam is metabolised in the oral mucosa by CYP3A4 and CYP3A5

**Fig. 6:**
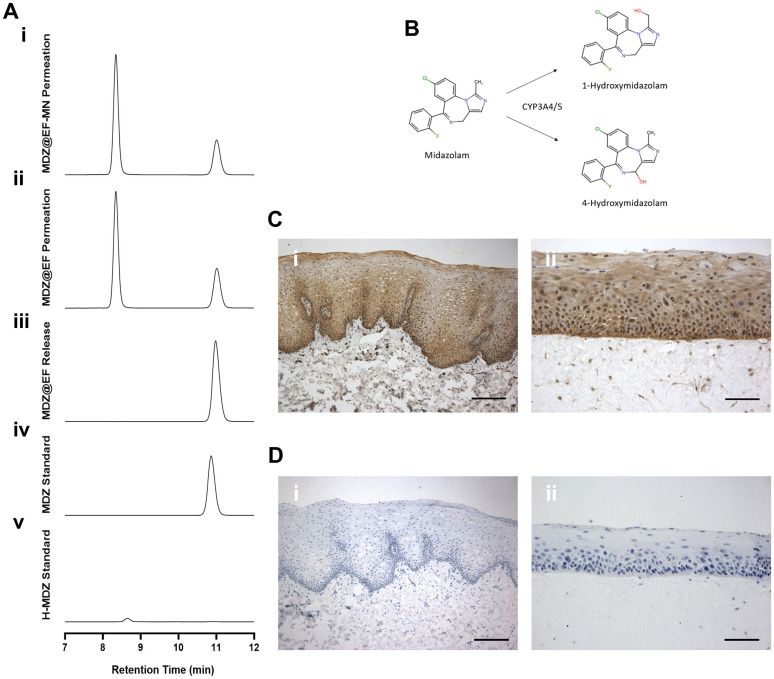
Midazolam is metabolised in the oral mucosal equivalents. Midazolam-incorporated membranes were manufactured from electrospun PVP (10%, w/w) and RS100 (12% w/w) with midazolam (5% w/w). Permeation of midazolam from mucoadhesive membranes across oral mucosal equivalents (OME) with (MDZ@EF-MN) (Ai) or without microneedles (MDZ@EF) (Aii) after 2 h using RP-HPLC compared to midazolam released directly into PBS (Aii) with midazolam and 1-hydroxymidazolam standards in PBS (Aiv and v) (*y*-axis are equal in all tests). Schematic diagram showing the xenobiotic metabolism of midazolam by CYP3A4/5 into the metabolites 1-hydroxymidazolam and 4-hydroxymedazolam (B). CYP3A4 immno-positive staining was identified by IHC in human buccal mucosa (Ci) and OME (Cii) with negative controls shown for both human buccal mucosa (Di) and OME (Dii). Scale bar = 200 µm (Ci and Di) and 100 µm in (Cii and Dii).

Following permeation of midazolam HCl from MDZ@EF or MDZ@EF-MN through OME, two peaks were observed on the HPLC chromatogram, at retention times of 8.5 and 10.5 minutes (Fig. 6Ai and ii), compared to just a single retention peak at 10.5 minutes for midazolam HCl released from the device directly into PBS (Fig. 6Aiii). The peak at 10.5 minutes corresponded to that of the midazolam HCl standard (Fig. 6Aiv), while the peak at 8.5 minutes is due to the presence of the midazolam HCl metabolite, 1-hydroxymidazolam (Fig. 6Av). After 2 h the levels of 1-hydroxymidazolam in the receptive medium was 81.3% for MDZ@EF and 86.2% for MDZ@EF-MN. For midazolam HCl 8.5% of drug was recovered from the receptive medium for MDZ@EF and 10.5% for MDZ@EF-MN, therefore total permeation (midazolam + 1-hydroxymidazolam) through the OME was 89.5% for MDZ@EF and 96.5% for MDZ@EF-MN, respectively. Midazolam is known to be metabolised by the xenobiotic iso-enzymes CYP3A4 and CYP3A5 to 1-hydroxymidazolam (Fig. 6B).^34^ Immunohistochemistry of human buccal mucosa (Fig. 6Ci) and OME (Fig. 6Cii) confirmed that CYP3A4 was abundant throughout the mucosal epithelium with highest levels in the basal keratinocytes. Control sections showed no background immunostaining (Fig. 6Di and ii).

### 3.6 F(ab)-loaded mucoadhesive electrospun fibres manufacture and characterisation

**Fig. 7:**
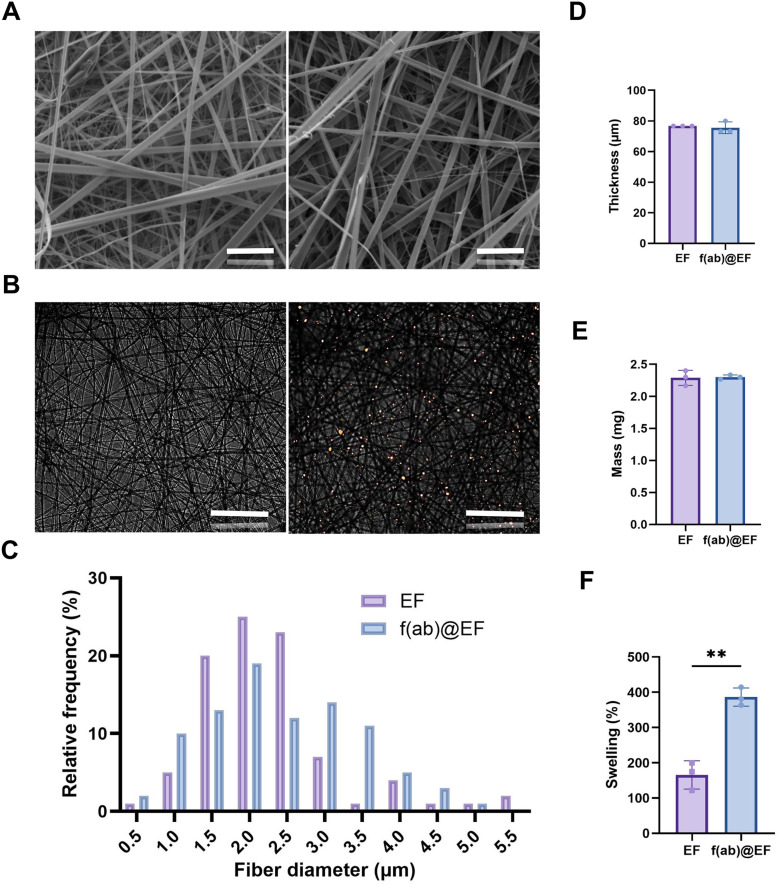
F(ab)-incorporated mucoadhesive electrospun fibre manufacture and characterisation. Electrospun fibres (EF) manufactured from electrospinning PVP (10% w/w) and RS100 (12% w/w) were produced to incorporate f(ab) (0.013% w/w). Representative SEM images of the (Ai) non-mediated EF and (Aii) f(ab)@EF revealed uniform fibres; scale bar = 20 µm (B) with fluorescent microscopy showing Texas Red labelled f(ab) distributed as an aggregate throughout the fibres, scale bar = 100 µm (C) with no difference in diameter between the two membranes. No significant difference in (D) thickness, (E) mass or (F) swelling properties were observed. Data are presented as mean ± SD with a statistically significant difference determined using a Student’'s *t* test where **p < 0.01; *n* = 3.

Following successful transmucosal permeation of midazolam from the MDZ@EF-MN, investigations were performed to deliver a biologic as a more challenging class of therapeutic, as high molecular mass molecules do not permeate the oral epithelium barrier. F(ab), as a model for monoclonal antibody therapy, were incorporated at 0.013% w/w and were found to be distributed as an aggregate throughout the fibres, with no observable difference in fibre diameter compared to non-medicated EF (Fig. 7A–C). The mean thickness of f(ab)@EF membranes was 75.6 ± 3.9 µm, compared to 76.67 ± 0.00 µm for non-medicated EF controls (Fig. 7D), with 10 mm ∅ f(ab)@EF membranes having a mean mass of 2.30 ± 0.03 mg that were not significantly different to the non-medicated EF (2.29 ± 0.12 mg) membranes (Fig. 7E). The final f(ab) content of 10 mm ∅ f(ab)@EF membranes was calculated as 298.66 ± 4.33 ng (equivalent to 3.8 ng mm^−2^ or 0.013% w/w). Following 2 h submersion in PBS, the mean swelling of f(ab)@EF was 400 ± 160%, significantly greater when compared to 165.5 ± 40.1% for non-medicated EF controls (*p* > 0.01) (Fig. 7F).

### 3.7 F(ab) delivered from the electrospun fibre-microneedle composite increases permeation through buccal mucosa

**Fig. 8:**
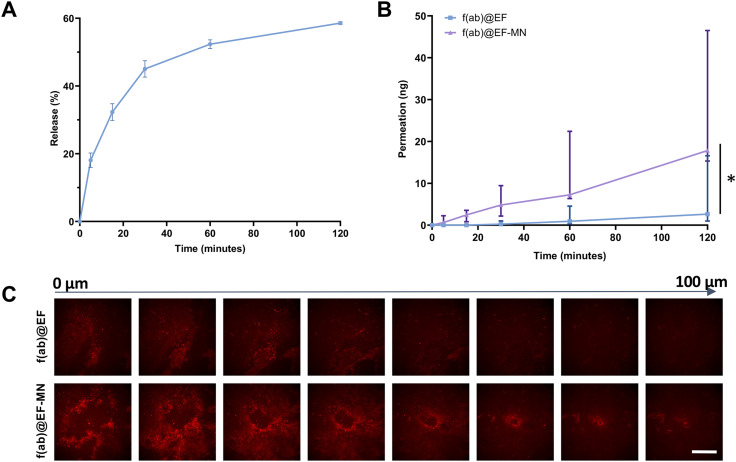
F(ab) delivery is enhanced using microneedle-mediated delivery. (A) f(ab) release from the electrospun membranes into PBS was measured over 2 h. Data presented as mean ± SD; *n* = 3. (B) f(ab) permeation (f(ab)@EF) through tissue-engineered oral mucosal equivalents was enhanced by microneedle-mediated (f(ab)@EF-MN) delivery. Data presented as median ± interquartile ranges with statistical significance determined using a Wilcoxon matched paired signed-rank test where *p < 0.05. (C) Permeation from f(ab)@EF-MN visualised overtime by fluorescent confocal microscopy compared to f(ab)@EF alone. Scale bar = 500 µm.

Initial f(ab) release into PBS was rapid from the f(ab)@EF with 31.9 ± 2.1% released within the first 30 minutes (Fig. 8A) which plateaued at 60 minutes with 57.9 ± 1.3% f(ab) released by 2 h. Permeation across OME was significantly increased from a median of 0.25 ng from the f(ab)@EF to 3.87 ng in the f(ab)@EF-MN (*p* < 0.05), when microneedles were present after 2 h. Permeation rates were also increased at short time periods, with 2.45 ng f(ab) detected following 15 minutes administration from the f(ab)@EF-MN construct but none detectable from EF alone, and 4.81 ng compared to 0.25 ng at 30 minutes (Fig. 8B). Penetration of Texas Red-conjugated f(ab) into the OME was visualised by confocal microscopy and confirmed that the microneedles increased permeation with increased fluorescence present in the epithelium surrounding a micropore and an increased depth of f(ab) penetration through the epithelium (Fig. 8C).

## 4. Discussion

Transmucosal drug delivery presents an attractive alternative to conventional administration routes. The oral mucosa provides a highly vascularised site with potential for rapid systemic absorption while bypassing premature metabolism through hepatic first-pass.^1^ The primary challenge remains the barrier properties of the epithelium, which limits drug permeation through the oral mucosa,^4^ coupled with limited residence times due to salivary washout.^35^ Medications intended for transmucosal delivery are limited, and there is a lack of suitable formulations being developed,^36^ leading to off-label drug administration and patient compliance issues.

Mucoadhesive electrospun patches have been developed to increase retention time to the oral mucosa,^37,38^ overcoming some drug delivery limitations by allowing improved drug contact time with the epithelium, increasing permeation opportunity.^9,10,18^ However, focus has remained on local drug delivery, failing to take advantage of the highly vascularised architecture of the oral cavity and the potential for rapid systemic delivery of drugs. Additionally, permeation of large molecules, such as biologics, cannot permeate the epithelial barrier at sufficient rate for systemic delivery.^18,39^ Here, we investigated the integration of electrospun PVP/Eudragit RS100 patches, as a versatile drug reservoir, with solid microneedles, offering controlled release whilst simultaneously mediating permeation across the epithelial barrier and improving the drug bioavailability of two compounds; midazolam HCl and antibody f(ab) fragments.

Originally developed for intradermal drug delivery, microneedles offer a minimally invasive approach to enhance drug permeation by creating micropores that penetrate through the epithelium to allow direct passage of drugs close to the blood vessels in the underlying connective tissue.^40^ PLA was chosen to manufacture solid microneedles, due to its known biocompatibility and widespread use in biomedical applications.^41^ OCT imaging confirmed that the microneedles create visible micropores penetrating through the oral epithelium to the lamina propria of porcine buccal tissue, confirming previous reports that PLA produces mechanically suitable microneedles, withstanding penetration forces equal or higher than oral mucosa.^42^ Additionally, we note that the solid state of PLA maintains the micropores within the oral mucosa for prolonged drug release, similar to that seen in dermal applications. Unlike drug loading *via* hollow, dissolvable or directly coated microneedles, which is limited by the microneedle dimensions and array size, coupling a membrane reservoir can be adapted to increase loading efficiency^29,30,43^ and, as demonstrated here, widens the number of drugs available for administration at therapeutic doses. Additionally, swelling properties of the patch can be tailored through polymer choice and fibre dimensions within the membrane reservoir, facilitating controlled release of poorly permeable drugs by maintaining enhanced drug bioavailability over prolonged periods.^12,44^ The combined mucoadhesive patch microneedle composite also improves rapid and firm adhesion to the oral mucosa. Previous reports have shown that a PVP/RS100 mucoadhesive patch adhered within seconds to the oral mucosa with adhesion lasting for approximately 2 h,^9^ consistent with our findings of 2.3 ± 0.6 h. However, the device developed in this study anchors the patch more firmly at the mucosal surface, remaining adherent for over 7 h, further improving residency times. This allows rapid and firm adhesion (conferring less risk of choking hazard for conditions such as status epilepticus) with simultaneous drug release for conditions that require rapid drug delivery, while also displaying prolonged adhesion for drugs that need sustained release over several hours.

Midazolam, a small molecule drug used in the treatment of seizures, is currently administered as an oromucosal solution, resulting in limited residency times within the oral mucosa. Re-administration is necessary every 5–10 minutes until the seizure subsides.^45^ This dosing regimen is subject to unquantifiable drug amounts, leading to increased risk of toxic side effects, and an unnecessarily prolonged wait time to reach adequate dosing. A study by McIntyre *et al.* showed that 44% of patients receiving buccal midazolam experienced suboptimal treatment with the inability to terminate the initial seizure within 10 minutes, without respiratory depression or recurrence within 1 h,^46^ highlighting a clinical need for improvement of benzodiazepine administration. Soroushnia *et al.*, developed a midazolam nanosuspension to enhance buccal administration to address bioavailability issues. Although they were able to demonstrate higher solubility, enhanced absorption and improved bioavailability compared to pure coarse midazolam powder, without a delivery device to maintain drug contact remains limited.^47^ To improve the delivery method, Onishi *et al.*, developed a mucoadhesive patch comprised of an outer Carbopol 934 region, central drug region containing diazepam dissolved in propylene glycol alone or propylene glycol containing oleic acid with a Tegaderm backing film.^48^ In an *in vivo* animal study, they found that the patch remained adhered until removal after 1 h and delivered a near pharmacologically effective plasma concentration for humans of 223 ng mL^−1^ at 10 minutes after administration. Our dual microneedle-mediated patch delivery addresses both concerns concurrently, resulting in a seven-fold increase in permeated midazolam at 15 minutes, compared to patch only delivery, demonstrating the potential of improving rapid delivery of emergency medication within a critical timeframe.

Here, we showed that midazolam was quickly metabolised within the OME. OME are increasingly being recognised as a physiologically relevant model to simulate the oral mucosa in drug toxicity^49^ and permeability testing,^50^ whilst offering a more ethical alternative to animal models and a more readily available, reproducible model that is representative of human tissue. It is also becoming increasingly acknowledged that the oral mucosa contains xenobiotic metabolising enzymes and that OME mirrors their expression.^51^ Midazolam undergoes hydroxylation by CYP3A4 to the major metabolite 1-hydroxymidazolam, and to a minor metabolite 4-hydroxymidazolam. These metabolites undergo further glucuronidation by UDP-glucuronosyltransferases (UGTs), mainly UGT1A4, to form 1-hydroxymidazolam glucuronide, which is excreted.^52^ We detected the presence of CYP3A4 enzymes within the OME, with the same expression patterns seen in native oral mucosa. There are conflicting reports on the expression of CYP3A4 in oral tissues, with some confirming the presence of CYP3A4 in oral biopsies,^53^ whilst others claim levels of its isoenzyme, CYP3A5 only.^54^ Relevant phase II UGTs have also been evidenced within the oral mucosa.^55^ The resultant major metabolite is 1-hydroxymidazolam, which has a ten times weaker affinity for the pharmacological receptor than midazolam. 1-Hydroxymidazolam accumulation is associated with prolonged sedation and renal failure in patients,^56^ demonstrating that the metabolite is clinically active, but its pharmacological impact compared to midazolam is debated.^57^ Re-administration to compensate for this metabolism can lead to further toxic side effects. Increased drug permeation rates through microneedle-mediated delivery could facilitate more rapid delivery to the lamina propria and avoidance of xenobiotic metabolism within the epithelium, increasing the bioavailability of clinically active drugs.

In recent years, antibody therapies have become an important class of therapeutics and by the end of 2024 the FDA had approved 159 antibody-based biologics,^58^ for treatment against a range of conditions including cancers,^59^ immune-related disease^60^ and infectious disease.^61^ Due to their smaller size, f(ab) have become a distinctive class of therapeutic molecules with four currently FDA approved.^58^ Structurally, these are large proteins (∼50 kDa) that remain susceptible to enzymatic degradation within the digestive tract.^62^ Due to their size, lack of permeability and predisposition to metabolism, f(ab) are typically administered *via* intravenous injection making them inaccessible to patients without trained personnel and undesirable to those with needle phobia. Systemic delivery also requires higher dosing, associated with off-target toxicity.^63^ Transmucosal delivery could offer a superior route for delivery. In pre-clinical studies, anti-TNF-alpha f(ab) delivered to OME demonstrated good efficacy in the local treatment of inflammatory-mediated oral ulcers.^18^ However, the research highlights the challenges of delivering biologics and large molecules across an epithelium for systemic delivery, as only approximately 5% of f(ab) had permeated the intact epithelium after 2 h.^18^ Here, we also saw limited f(ab) permeation after 2 h administration of f(ab)-loaded patches to OME, however with the addition of microneedles, this was increased a 15-fold, effectively reducing administration time by over seven h and offers the first report of f(ab) fragments successfully being delivered across an intact epithelium into the underlying lamina propria.

In summary, this research demonstrates the feasibility and advantages of microneedle-mediated transmucosal drug delivery, offering a minimally invasive, patient-friendly alternative that is superior to traditional administration methods. The use of microneedles enhances drug permeation by facilitating passage of drugs – particularly large molecules that are poorly permeable – through the epithelial barrier and/or to increase the rate of delivery. Combining microneedles with an electrospun mucoadhesive patch was successful in overcoming reported challenges in the transmucosal field, specifically poor residency times, limitations on permeation, and dosing limitations with alternative microneedle designs. This design enhanced drug permeation of two very different molecules, providing a rational design for further therapeutic incorporation. This unique approach combines the benefits of microneedles and electrospinning as an effective method for improving drug bioavailability and therapeutic outcomes, representing a highly promising advancement in transmucosal drug administration.

## Author contributions

Cerys Berry: methodology, investigation, formal analysis, writing – original draft preparation, writing – review & editing. Jake G. Edmans: methodology, investigation, writing – review & editing. Klaudia M. Slowik: methodology, investigation, writing – review & editing. Robert A Byers: methodology, investigation, writing – review & editing. Simon Danby: methodology, writing – review & editing. Paul V. Hatton: conceptualization, supervision, writing – review & editing, funding acquisition. Craig Murdoch: conceptualization, supervision, methodology, writing – review & editing. Helen E. Colley: conceptualization, methodology, supervision, project administration, writing – original draft preparation, writing – review & editing, funding acquisition.

## Conflicts of interest

The authors declare no conflicts of interest.

## Data Availability

Data are available upon request from the authors.
